# Acute kidney injury after out-of-hospital cardiac arrest

**DOI:** 10.1186/s13054-024-04936-w

**Published:** 2024-05-18

**Authors:** Karoline Korsholm Jeppesen, Sebastian Buhl Rasmussen, Jesper Kjaergaard, Henrik Schmidt, Simon Mølstrøm, Rasmus Paulin Beske, Johannes Grand, Hanne Berg Ravn, Matilde Winther-Jensen, Martin Abild Stengaard Meyer, Christian Hassager, Jacob Eifer Møller

**Affiliations:** 1 Department of Cardiology, Odense University Hospital, J. B. Winsloews Vej 4, 5000 Odense C, Denmark; 2 Department of Anesthesiology and Intensive Care, Odense University Hospital, Odense, Denmark; 3 Department of Cardiology, The Heart Center, Copenhagen University Hospital Rigshospitalet, Copenhagen, Denmark; 4 Department of Clinical Medicine, University of Copenhagen, Copenhagen, Denmark; 5 Department of Clinical Research, University of Southern Denmark, Odense, Denmark

**Keywords:** Post-cardiac arrest, Cardiopulmonary resuscitation, Acute kidney injury, Continuous kidney replacement therapy

## Abstract

**Background:**

Acute kidney injury (AKI) is a significant risk factor associated with reduced survival following out-of-hospital cardiac arrest (OHCA). Whether the severity of AKI simply serves as a surrogate measure of worse peri-arrest conditions, or represents an additional risk to long-term survival remains unclear.

**Methods:**

This is a sub-study derived from a randomized trial in which 789 comatose adult OHCA patients with presumed cardiac cause and sustained return of spontaneous circulation (ROSC) were enrolled. Patients without prior dialysis dependent kidney disease and surviving at least 48 h were included (N = 759). AKI was defined by the kidney disease: improving global outcome (KDIGO) classification, and patients were divided into groups based on the development of AKI and the need for continuous kidney replacement therapy (CKRT), thus establishing three groups of patients—No AKI, AKI no CKRT, and AKI CKRT. Primary outcome was overall survival within 365 days after OHCA according to AKI group. Adjusted Cox proportional hazard models were used to assess overall survival within 365 days according to the three groups.

**Results:**

In the whole population, median age was 64 (54–73) years, 80% male, 90% of patients presented with shockable rhythm, and time to ROSC was median 18 (12–26) min. A total of 254 (33.5%) patients developed AKI according to the KDIGO definition, with 77 requiring CKRT and 177 without need for CKRT. AKI CKRT patients had longer time-to-ROSC and worse metabolic derangement at hospital admission. Overall survival within 365 days from OHCA decreased with the severity of kidney injury. Adjusted Cox regression analysis found that AKI, both with and without CKRT, was significantly associated with reduced overall survival up until 365 days, with comparable hazard ratios relative to no AKI (HR 1.75, 95% CI 1.13–2.70 vs. HR 1.76, 95% CI 1.30–2.39).

**Conclusions:**

In comatose patients who had been resuscitated after OHCA, patients developing AKI, with or without initiation of CKRT, had a worse 1-year overall survival compared to non-AKI patients. This association remains statistically significant after adjusting for other peri-arrest risk factors.

*Trial registration*: The BOX trial is registered at ClinicalTrials.gov: NCT03141099.

**Supplementary Information:**

The online version contains supplementary material available at 10.1186/s13054-024-04936-w.

## Introduction

Acute kidney injury (AKI) is a serious complication occurring in around 40% of patients resuscitated after out-of-hospital cardiac arrest (OHCA) and is associated with worse short- and long-term survival [1, 2]. Known risk factors for AKI includes prolonged time to return of spontaneous circulation (ROSC), initial non-shockable rhythm, and post-resuscitation shock [3]. AKI was previously regarded as primarily a consequence of unfavorable peri-arrest conditions [4]. However, several studies have demonstrated that AKI may have an impact on survival itself [1, 2, 5–10]. Additionally, patients presenting with preexisting kidney impairment prior to the OHCA event face an increased 90-day mortality [11]. Therefore, post-resuscitation care to attenuate further kidney impairment is important and may lead to a better outcome.

Patients resuscitated after cardiac arrest may develop post-cardiac arrest syndrome (PCAS)—which encompasses post-ischemic neurological dysfunction, myocardial stunning, systemic inflammatory response syndrome, and impaired vasoregulation [12, 13]. The latter may result in low cardiac output syndrome with vasoplegic shock and ultimately poor end-organ perfusion [14]. Management consists of delicate balance of vasopressors, inotropes, fluid management, and diuretics. However, evidence on kidney protective targets is currently limited and the pathophysiology leading to AKI is not fully understood. Furthermore, it is crucial to acknowledge that the diagnosis of AKI encompasses various causes and conditions rather than representing a specific disease. Consequently, there may be prognostic differences when adjusting for individual risk factors and categorizing according to AKI severity.

The objective of the present study was to determine characteristics associated with AKI according to the Kidney Disease Improving Global Outcomes (KDIGO) classification [15] and the prognostic importance of AKI severity in a well-defined OHCA population enrolled in the Blood Pressure and Oxygenation Targets After OHCA (BOX) trial [16, 17].

## Methods

### The BOX trial

The BOX trial (NCT03141099) was an investigator-initiated, randomized trial with a two-by-two factorial design where comatose OHCA patients were enrolled at 2 tertiary cardiac arrest centers in Denmark between March 10th, 2017, and December 11th, 2021. Trial methods and the primary outcome results are described elsewhere [16, 17]. Briefly, adult patients (≥ 18 years) resuscitated after OHCA of presumed cardiac cause and that remained comatose after sustained ROSC were randomly assigned to a mean arterial pressure (MAP) treatment target of either 63 mmHg or 77 mmHg, and simultaneously randomized to open-label intervention of either restrictive (9–10 kPa) or liberal (13–14 kPa) oxygen treatment target [16, 17]. In addition, patients were randomized to device-based temperature intervention targeting 36 °C for 24 h followed by either a target of 37 °C for 12 or 48 h [18]. The original two studies from the BOX trial evaluated AKI requiring CKRT as an adverse event and found no difference between targets of MAP or oxygen treatment [16, 17]. Therefore, the data analysis in this sub-study was performed across the intervention groups.

### Treatment protocol

All patients included in the trial underwent target temperature management (TTM) as described above. During TTM patients were sedated (propofol and fentanyl) and mechanically ventilated.

In the first 24 h, a positive volume balance of approximately 1000 ml and diuresis of 1–1.5 ml/kg/h was targeted. Furosemide was administered as a first-line drug to increase diuresis if needed. CKRT was initiated at the discretion of treating physicians based on common indications for CKRT including oliguria or anuria, refractory volume overload, severe metabolic acidosis, electrolyte disturbances, or manifest uremic symptoms.

Plasma creatinine was measured upon hospital admission, and henceforward daily in the intensive care unit. If multiple plasma creatinine measurements were available the highest value was used for AKI assessment. Plasma creatinine ratio was calculated as the ratio of plasma creatinine at 48 h and the value at admission. Estimated glomerular filtration rate (eGFR) was determined upon hospital admission using the CKD-EPI 2021 formula [19]. Collected urine output was recorded at 24- and 48 h. Urine creatinine clearance was calculated at 48 h based on 24-h urine creatinine and volume, and plasma-creatinine. Thus, only patients surviving beyond 48 h were included in the analysis to ensure the possibility to record kidney injury. Furthermore, patients receiving chronic dialysis before OHCA were excluded from analysis.

AKI was defined by the Kidney Disease: Improving Global Outcome (KDIGO) classification [15] (Table S1, Supplemental Material), and patients were divided into groups based on development of AKI and need for CKRT, thus establishing three groups of patients—No AKI, AKI no CKRT and AKI CKRT.

### Endpoints

The primary endpoint of this study was overall survival within 365 days after OHCA, stratified according to patients with no AKI, AKI no CKRT, and AKI CKRT. Furthermore, the characteristics associated with development of AKI after OHCA were assessed. Lastly, we assessed the need for intermittent or chronic dialysis at 3 months and 1 year post-discharge in patients who underwent CKRT within 5 days of their ICU stay.

### Statistical analysis

The primary study’s sample size calculation determined the size of the study population in this pre-specified sub-study. Survival probabilities were calculated using the Kaplan–Meier estimator for No AKI, AKI no CKRT, and AKI CKRT, respectively. The Log-Rank test was used to test for statistically significant differences between groups.

Cox proportional hazard models were used to assess overall survival within 365 days from hospital admission for No AKI, AKI no CKRT, and AKI CKRT, respectively. The corresponding hazard ratios (HRs) and 95% confidence intervals (CIs) were listed with No AKI as the reference. The proportional hazards assumption and linearity on the log-hazard scale were assessed using cumulative martingale residuals. The Cox proportional hazard models were adjusted for the following confounders; age, sex, eGFR upon admission, diabetes mellitus, heart failure, witnessed arrest, bystander cardio-pulmonary resuscitation, shockable rhythm, time-to-ROSC, and acute percutaneous coronary intervention.

A two-sided *p*-value < 0.05 was considered statistically significant. All analyses were conducted in R Statistical Software version 6.1.524 (Posit team 2023).

## Results

The BOX trial enrolled 802 patients between March 2017 and December 2021. Consent was declined in 12 patients and 1 patient was randomized twice, hence, the intention-to-treat analysis included 789 patients [16, 17].

In the present sub-study, we further excluded patients receiving chronic dialysis prior to admission and patients who died within 48 h of admission. Thus 759 patients underwent analysis (Figure S1, Supplemental Material). Baseline characteristics for patients with No AKI, AKI no CKRT, and AKI CKRT are presented in Table 1. The population consisted primarily of men around 60 years of age and cardiovascular comorbidities were common. Majority of patients had a witnessed cardiac arrest with bystander cardio-pulmonary resuscitation and an initial shockable rhythm.

**Table 1:** Patient demographics and clinical characteristics

	No AKI (N = 505)	AKI no CKRT (N = 177)	AKI CKRT (N = 77)	*p* value
*Patient characteristics*				
Age, years*	62 (53–71)	67 (56–74)	67 (55–73)	***0.006***
Male sex^**†**^	400 (79%)	150 (85%)	65 (84%)	0.21
*Previous medical history*				
Hypertension^**‡**^	226 (45%)	84 (48%)	36 (47%)	0.79
Diabetes^**§**^	63 (13%)	30 (17%)	12 (16%)	0.29
Ischemic heart disease^**||**^	109 (22%)	27 (15%)	27 (35%)	***0.001***
Atrial fibrillation^**#**^	72 (14%)	30 (17%)	16 (21%)	0.29
Heart failure**	79 (16%)	29 (16%)	21 (27%)	***0.040***
Stroke^**††**^	40 (8%)	10 (6%)	7 (9%)	0.52
Chronic kidney disease^**‡‡**^	16 (3%)	9 (5%)	8 (10%)	***0.013***
*Characteristics of cardiac arrest*				
Shockable rhythm^**§§**^	456 (91%)	152 (86%)	72 (94%)	0.11
Witnessed arrest^**||||**^	426 (85%)	156 (88%)	61 (79%)	0.18
Bystander CPR^**##**^	441 (89%)	152 (87%)	64 (83%)	0.28
Time-to-ROSC, minutes***	15 (11–23)	21 (15–30)	30 (19–45)	** < *0.001***
*Findings and procedures at hospital arrival*				
pH on arrival^**†††**^	7.24 ± 0.11	7.20 ± 0.12	7.07 ± 0.16	** < *0.001***
Lactate on arrival, mmol/L^**‡‡‡**^	4.3 (2.5–7.1)	5.5 (3.4–8.2)	8.4 (6.5–12.5)	** < *0.001***
First recorded base excess, mmol/L^**§§§**^	− 6.3 (− 9.4 to − 3.5)	− 6.9 (− 10.9 to − 4.0)	− 13 (− 17.2 to − 9.7	** < *0.001***
First recorded PaO_2_, kPa^**||||**||^	10.7 (6.5–21.2)	11.2 (7.2–20.1)	9.1 (7.2–12.5)	0.19
First recorded PaCO_2_, kPa^**###**^	6.2 (5.5–7.0)	6.6 (5.9–7.6)	7.6 (6.0–9.0)	** < *0.001***
STEMI****	228 (46%)	76 (44%)	30 (39%)	0.52
Immediate coronary angiography^**††††**^	460 (91%)	165 (93%)	68 (88%)	0.42
Percutaneous coronary intervention^**‡‡‡‡**^	206 (45%)	77 (47%)	34 (50%)	0.70
*Interventional characteristics*				
PaO_2_ during ICU, kPa^**§§§§**^	12.6 (11.4–13.8)	12.4 (10.9–13.9)	12.3 (11.2–13.4)	0.38
MAP during ICU, mmHg^**||||||||**^	78.4 ± 8.8	76.7 ± 9.45	73.2 ± 9.1	** < *0.001***
Temperature first 72 h, °C^####^	36.8 (36.5–37.0)	36.7 (36.5–37.0)	36.7 (36.3–37.0)	0.18

Continuous data are expressed as mean (± standard deviation) for parametric data and median (25th–75th percentiles) for non-parametric data. Categorical variables are expressed as counts (percentage). The values of the interventional characteristics were derived by calculating weighted means, accounting for variations in measurement frequency during ICU, thus ensuring each day contributed proportionally to the overall means. Bold itlaic values indicate highligth statistical significance. Abbreviations: *CKRT;* continuous kidney replacement therapy, *CPR*; cardio-pulmonary resuscitation, *ICU*; intensive care unit, *MAP*; mean arterial pressure, *PaCO*_*2*_; partial pressure of arterial carbon dioxide*, PaO*_*2*_; partial pressure of arterial oxygen, *ROSC;* return of spontaneous circulation, *STEMI*; ST-segment elevation myocardial infarction

Missing data; *2 patients (0.3%), †1 patient (0.1%), ‡1 patient (0.1%), §2 patients (0.3%), ||2 patients (0.3%), #3 patients(0.4%), **2 patients (0.3%), ††1 patient (0.1%), ‡‡1 patient (0.1%), §§1 patient (0.1%), ||||1 patient (0.1%), ##12 patients (1.6%), ***17 patients (2.2%), †††48 patients (6.3%), ‡‡‡22 patients (2.9%), §§§25 patients (3.3%), ||||||37 patients (4.8%), ###90 patients (11.9%),****13 patients (1.7%),††††13 patients (1.7%),‡‡‡‡68 patients (9.0%), §§§§4 patients (0.5%), ||||||||3 patients (0.4%), ####15 patients (1.9%)

### Acute kidney injury

**Fig. 1:**
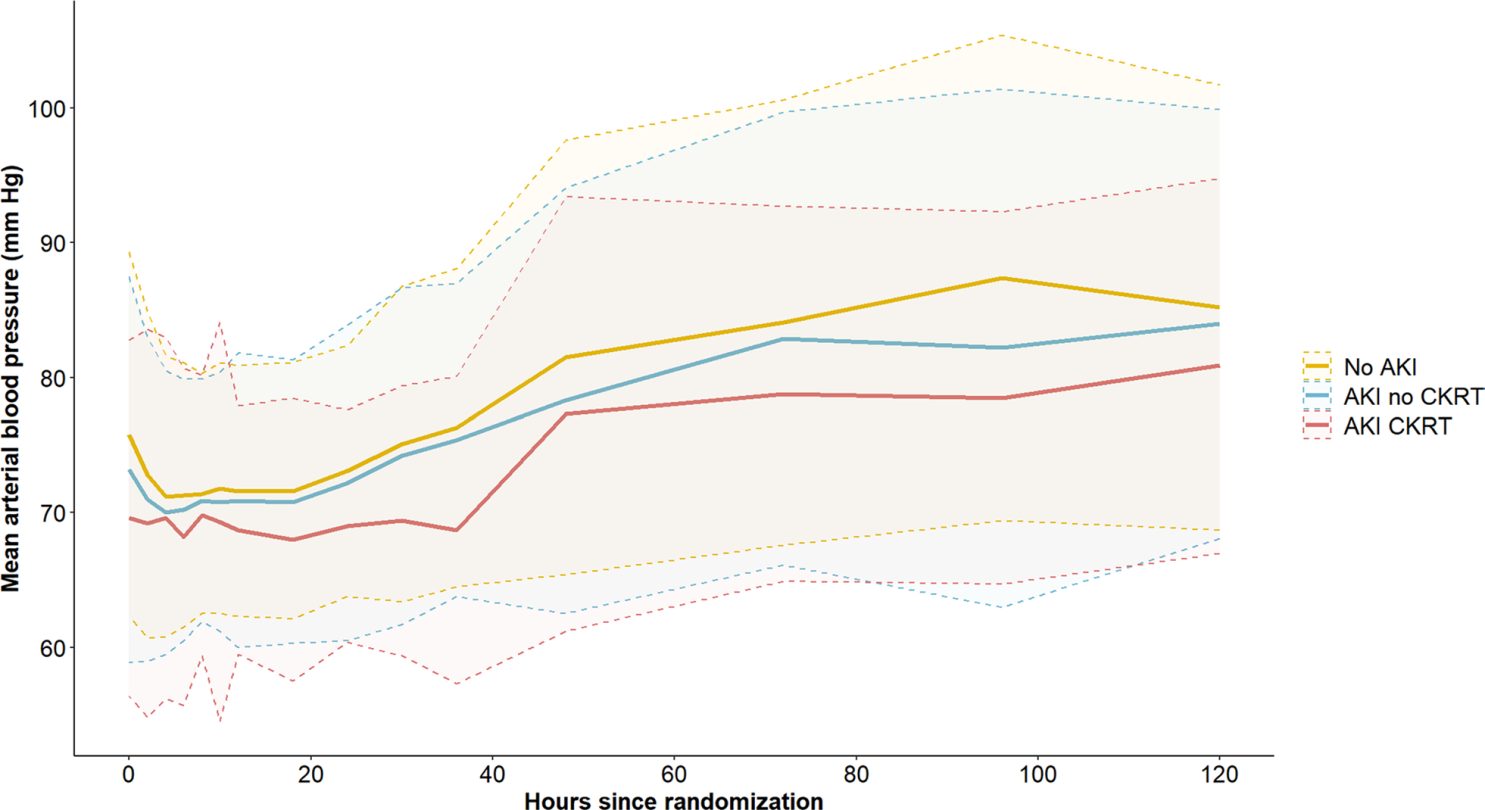
Mean arterial pressure. Mean arterial pressure (± standard deviation) over time according to group—No AKI, AKI no CKRT and AKI CKRT. *AKI*; acute kidney injury, *CKRT*; continuous kidney replacement therapy

**Fig. 2:**
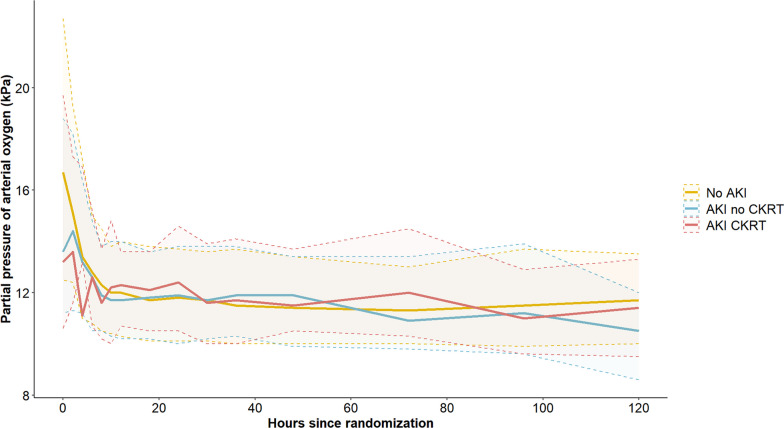
Partial pressure of arterial oxygen. Median partial pressure of arterial oxygen (25th–75th percentile) over time according to group—No AKI, AKI no CKRT, AKI CKRT. *AKI*; acute kidney injury, *CKRT*; continuous kidney replacement therapy

A total of 254 (33.5%) patients developed AKI according to the KDIGO definition, 177 without the need for CKRT and 77 with the need for CKRT. Patients developing AKI were generally older and had longer time-to-ROSC and correspondingly more severe metabolic derangement at hospital admission (Table 1). Values of MAP and PaO_2_ during the first 5 days of ICU are illustrated for AKI groups in Figs. 1 and 2. Significantly lower weighted mean values of MAP, extending up to ICU day 5, were observed in AKI patients and demonstrated a declining trend with increasing AKI severity (Table 1). Conversely, mean PaO_2_ and temperature exhibited comparable levels between groups (Table 1). Patients who developed AKI had a statistically significantly lower mean perfusion pressure during the period 6–12 h after admission to the ICU, but not at other time points (Figure S2, Table S2, Supplemental Material).

**Fig. 3:**
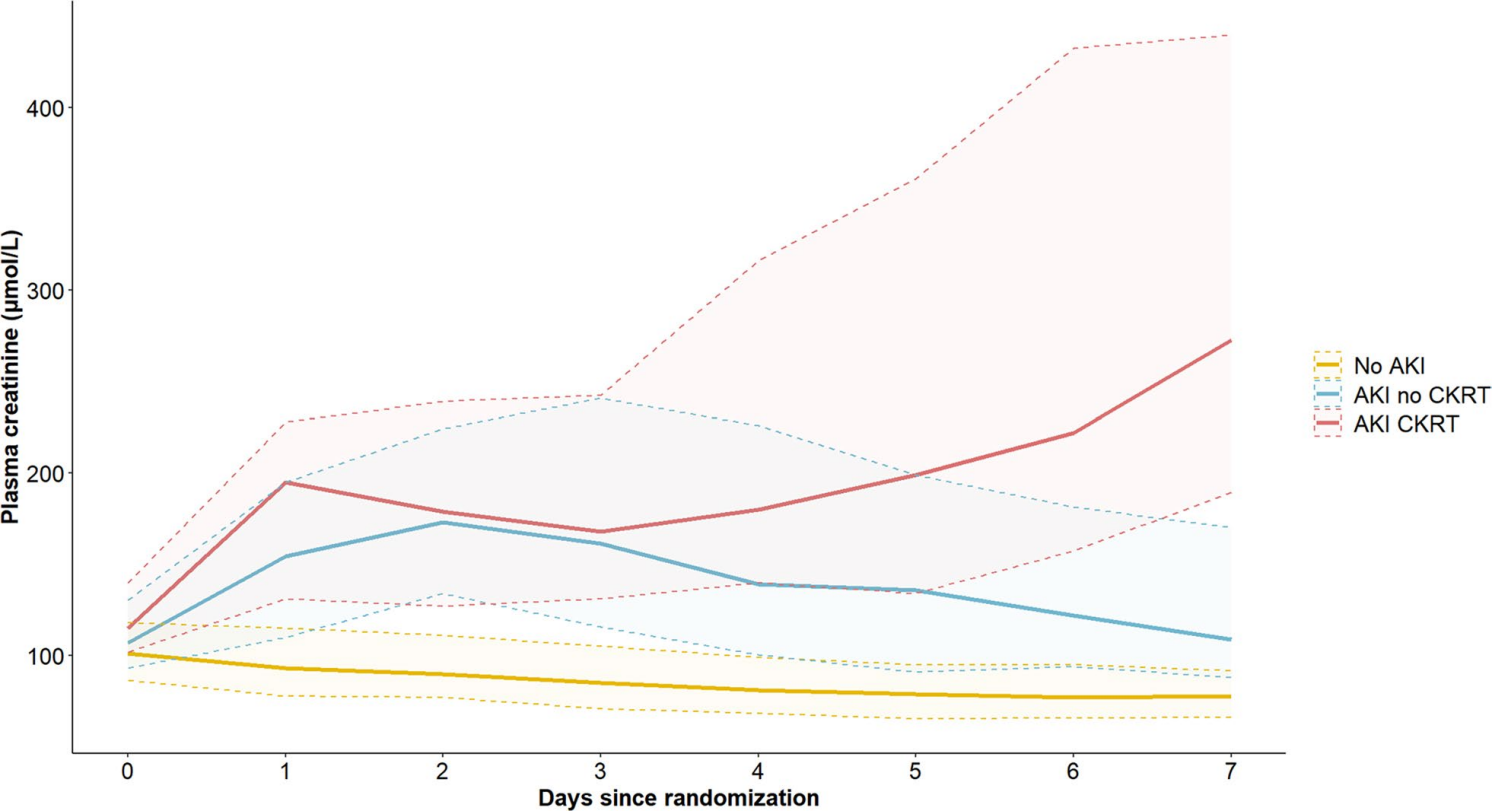
Plasma-creatinine. Median plasma-creatinine (25th–75th percentile) over time according to group—No AKI, AKI no CKRT, AKI CKRT. *AKI*; acute kidney injury, *CKRT*; continuous kidney replacement therapy

Blood samples and clinical markers of kidney function are listed in Table 2. The eGFR at hospital admission was inversely related to the severity of AKI (Table 2). Furthermore, both a low urine output and increasing plasma creatinine were seen in the AKI CKRT group, while AKI diagnosis was primarily driven by an increase in creatinine in the AKI no CKRT group (Table 2). The levels of plasma creatinine within the first 7 days of hospitalization according to each group are presented in Fig. 3.

**Table 2:** Kidney injury parameters

	No AKI (N = 505)	AKI no CKRT (N = 177)	AKI CKRT (N = 77)
eGFR on admission, ml/min/1.73 m^2^	69.8 ± 19.1	63.4 ± 18.5	57.5 ± 16.9
P-creatinine on admission, µmol/L	101 (87–118)	107 (93–131)	115 (102–140)
P-creatinine at 24 h, µmol/L	93 (78–115)	155 (110–195)	195 (132–228)
P-creatinine at 48 h, µmol/L	90 (77–111)	173 (134–224)	179 (127–239)
P-creatinine ratio*	0.91 (0.79–1.02)	1.51 (1.30–1.93)	1.49 (1.02–2.19)
Urine output day 1, ml/kg/h	1.32 (0.93–1.71)	1.15 (0.80–1.52)	0.28 (0.10–0.71)
Urine output day 2, ml/kg/h	1.38 (1.08–1.67)	1.21 (0.53–1.69)	0.34 (0.09–1.27)

Continuous data are expressed as mean (± standard deviation) for parametric data and median (25th–75th percentiles) for non-parametric data

*CKRT*, continuous kidney replacement therapy; eGFR, estimated glomerular filtration rate calculated according to CKD-EPI 2021 formula [19]

*P-creatinine ratio; the ratio of p-creatinine at 48 h and at admission

Among patients receiving CKRT within the first 5 ICU days, no survivors required intermittent or chronic dialysis at 3 months or 1 year after discharge.

### Survival analysis

**Fig. 4:**
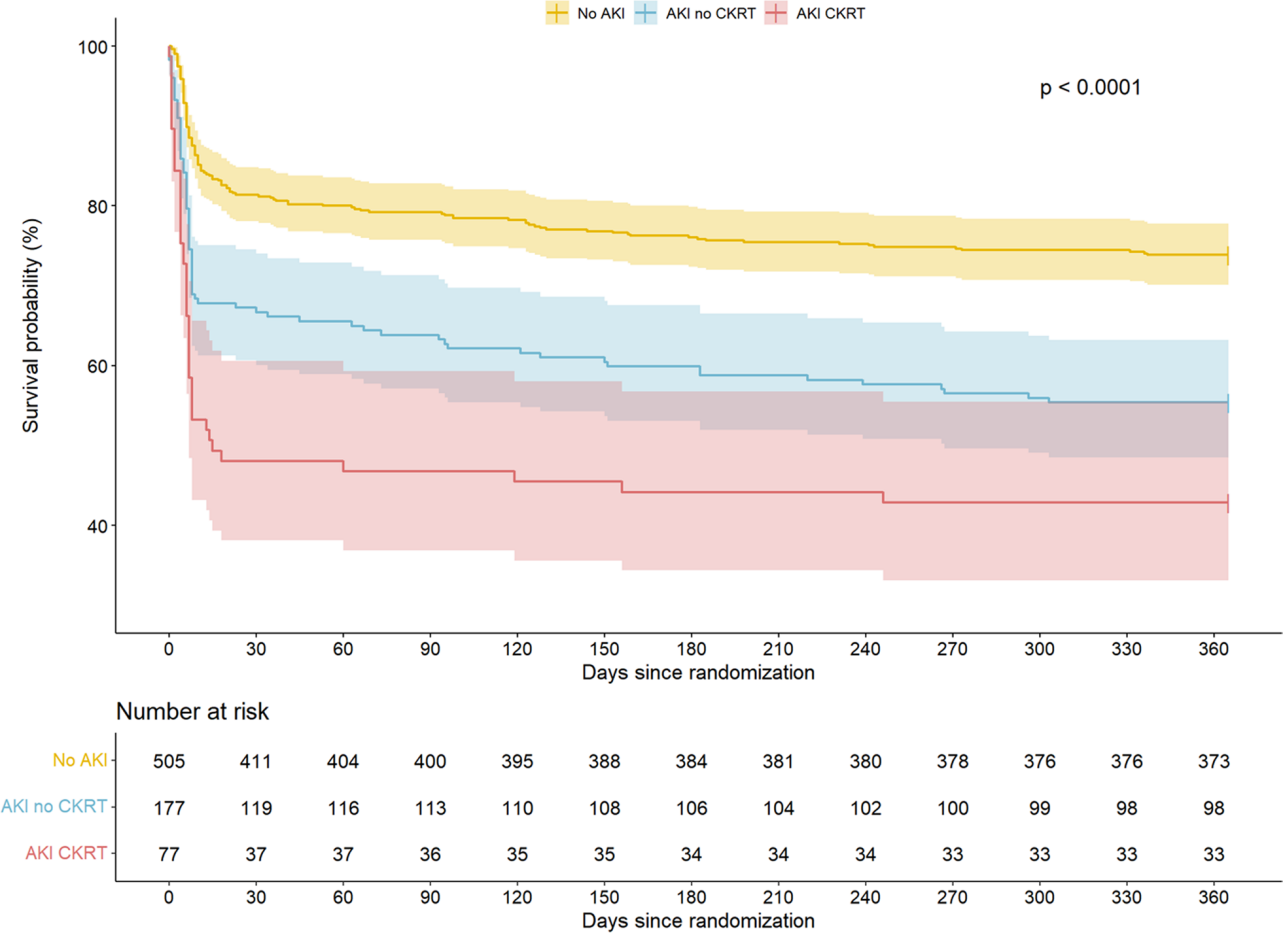
Kaplan Meier survival analysis. Kaplan Meier survival analysis displaying survival functions for No AKI, AKI no CKRT, and AKI CKRT. *AKI*; acute kidney injury, *CKRT*; continuous kidney replacement therapy

Kaplan–Meier survival estimates according to No AKI, AKI no CKRT, and AKI CKRT, respectively, are illustrated in Fig. 4 and demonstrate a statistically significant difference in survival between groups within 365 days after OHCA (Log-Rank *p* < 0.0001). Overall survival 365 days after hospital admission was 74% in patients with no AKI, 55% in patients with AKI and no CKRT, and 43% in the AKI CKRT group.

In unadjusted cox regression, AKI no CKRT (HR 1.99, 95% CI 1.51–2.63; *p* < 0.001) and AKI CKRT (HR 3.10, 95% CI 2.20–4.36; *p* < 0.001) were associated with increased hazard for death from all causes relative to patients without AKI (Table 3). After adjusting for age, sex, eGFR upon admission, diabetes mellitus, heart failure, witnessed arrest, bystander cardio-pulmonary resuscitation, shockable rhythm, time-to-ROSC, and acute percutaneous coronary intervention, both AKI with and without CKRT remained significantly associated with reduced overall survival after 365 days (Table 3). Notably, the hazard ratios for AKI with and without CKRT were comparable relative to no AKI (Table 3).

**Table 3:** Cox regression analysis

Covariate	All-cause mortality		
	HR	95% CI	*p* value
*Unadjusted model*			
AKI no CKRT (*v* no AKI)	1.99	1.51 to 2.63	** < *0.001***
AKI CKRT (*v* no AKI)	3.10	2.20 to 4.36	** < *0.001***
*Adjusted model*			
AKI no CKRT (*v* no AKI)	1.76	1.30 to 2.39	** < *0.001***
AKI CKRT (*v* no AKI)	1.75	1.13 to 2.70	***0.012***
Age (per year)	1.03	1.02 to 1.04	** < *0.001***
Sex (female *v* male)	1.61	1.17 to 2.20	***0.030***
eGFR upon admission (per ml/min/1.73 m^2^)	1.00	0.99 to 1.01	*0.71*
Diabetes mellitus (yes *v* no)	1.01	0.71 to 1.43	*0.95*
Heart failure (yes *v* no)	1.33	0.94 to 1.87	*0.10*
Shockable rhythm (yes *v* no)	0.49	0.34 to 0.70	** < *0.001***
Witnessed arrest (yes *v* no)	0.79	0.55 to 1.13	*0.20*
Bystander CPR (yes *v* no)	0.49	0.35 to 0.68	** < *0.001***
Time-to-ROSC (per minute)	1.02	1.02 to 1.03	** < *0.001***
Percutaneous coronary intervention (yes vs no)	0.96	0.73 to 1.27	*0.78*

Cox regression analysis evaluating risk of death within 365 days according to No AKI, AKI no CKRT, and AKI CKRT. Bold italic values indicate highligth statistical significance

*AKI*, acute kidney injury; *CI*, confidence interval; *CKRT*, continuous kidney replacement therapy; *CPR*, cardio-pulmonary resuscitation; *eGFR*, estimated glomerular filtration rate; *HR*, hazard ratio; *ROSC*, return of spontaneous circulation

## Discussion

In the present study, including resuscitated comatose OHCA patients of presumed cardiac cause, patients developing AKI requiring CKRT had longer time-to-ROSC and correspondingly more severe metabolic derangement at hospital admission than AKI patients without CKRT or without AKI. Patients with AKI, with or without CKRT, had a worse 1-year overall survival compared with patients without AKI. Following adjustment for peri-arrest parameters, AKI remained a significant risk factor for reduced overall survival, irrespective of CKRT initiation.

Our results add to current knowledge regarding predictors of AKI in OHCA patients, and the long-term consequences of having an episode of AKI within the post-resuscitation period. Thirty percent of patients developing AKI received CKRT in our study. This is in line with the observed frequency in a recent systematic review on cardiac arrest survivors [2]. Despite the frequent use of CKRT, several large randomized trials have found no survival benefit from especially early and aggressive use of CKRT in critically ill patients [20–22]. Similarly, a recent study on cardiac arrest patients found no difference in in-hospital survival between patients with AKI requiring CKRT within 72 h of admission and patients without the need for CKRT [23]. Looking at the characteristics of CKRT patients, it seems that there is an gradual increase in plasma creatinine from day 3 to day 7 (Fig. 3). Considering that CKRT was initiated in 70% of patients by 48 h and that the duration of CKRT was less than 3 days in more than half of the patients, it is likely that the delayed increase in creatinine represents patients weaned off CKRT with re-established water excretion, but still lacking normal renal clearance (Table S3 and S4, Supplemental Material). However, it seems that there are no long-term adverse clearance issues, as none of the patients who were on CKRT by day 5 required dialysis up to 1 year after discharge (Fig. 4).

In this study, we found a 12.5 percentage point lower 1-year survival among AKI patients receiving CKRT compared to AKI patients without CKRT. This difference in survival was even more pronounced when comparing AKI CKRT patients with patients without AKI (31 percentage point difference). The main divergence in survival was related to differences in short-term mortality, particularly within the initial 10 days after hospital admission (Table S8, Supplemental Material). Therefore, the main question remains whether severity of AKI is just a surrogate measure of worse peri-arrest conditions, or whether it is a significant risk factor for long-term survival in itself.

We identified increasing age, female sex, and particularly, time-to-ROSC as significantly associated with worse overall survival through adjusted Cox regression analysis. Conversely, having an initial shockable rhythm and receiving bystander cardiopulmonary resuscitation were significantly associated with a better outcome.

In alignment with the well-established cardiorenal syndrome, we observed a higher prevalence of cardiac pre-arrest comorbidities, such as ischemic heart disease and heart failure, within the AKI CKRT group (Table 1). Heart failure is known to trigger compensatory mechanisms, leading to fluid retention, venous congestion, and ultimately reduced kidney perfusion [24]. Furthermore, in OHCA patients with pre-existing cardiac dysfunction, a vicious cycle may be initiated due to a combination of hypoperfusion, ischemia–reperfusion injury, use of radiocontrast, positive pressure ventilation, etc. However, surprisingly, pre-existing heart failure did not emerge as a significant risk factor in the adjusted Cox analysis.

Patients with AKI requiring CKRT and AKI without CKRT were both associated with worse overall survival, but interestingly, following adjustment for known confounders, the hazards were similar between groups. Although the Cox regression analysis was adjusted for eGFR upon admission, some residual hazard may still be attributed to pre-admission kidney vulnerability in AKI patients, given the well-established association of chronic kidney impairment with cardiovascular death [25]. In this study, we found a gradually lower eGFR at baseline according to the severity of kidney injury (Table 2). This is in line with Tamura et al., who also found a graded association between lower eGFR at admission and a worse 3-month survival [11]. The variation in drug metabolism and clearance among AKI patients is recognized to influence treatment responses, potentially leading to an ominous outcome [26]. Given that preexisting kidney dysfunction is a risk factor for AKI, overall survival may be jeopardized due to a combination of cardiovascular dysfunction and altered pharmacokinetics [27].

Thus, regardless of severity, AKI occurrence is associated with worse overall survival, emphasizing the need for evidence-based post-resuscitation kidney-protective care. International post-resuscitation guidelines recommend a target of MAP > 65 mmHg, but based on sparse clinical evidence [28]. However, previous studies in OHCA have suggested a potential benefit of a further increase in MAP on kidney function [6, 29]. In this present study, we observed a significant association between lower MAP during the initial 5 days of ICU admission (Table 1) and lower perfusions pressure during the 6–24 h of ICU admission (Table S2, Supplemental Material), according to the severity of AKI. This association persisted, even though AKI CKRT patients had a significantly increased Vasoactive Inotropic Score (VIS) throughout the first 72 h (Table S5, Supplemental Material). Other studies have also demonstrated higher use of vasopressors to be associated with AKI severity [30, 31]. However, what at first glance may seem as a paradox or conflicting results, may be reconciled if certain phenotypes are more susceptible to lower perfusion pressure or if the association is the other way around, that patients with more severe organ injury require more inotropic support, increased filling pressures, and lower perfusion pressure. In this case, the lower MAP is not the cause but a consequence of the patient's condition.

Nevertheless, when evaluating observational data, it is important to cautiously assess the use of MAP as a surrogate measure for organ perfusion pressure. One study found a low MAP, but not a low cardiac output, to be associated with AKI development in OHCA patients [32].

To effectively control for potential confounders, it is essential to prioritize kidney protective endpoints in randomized clinical trials. We recently demonstrated that patients randomized to a combination of a low mean arterial blood-pressure (63 mmHg) and a liberal oxygen target (13–14 kPa) had a significantly increased risk of mild-stage AKI [33]. This is especially important in light of the results of the present study, which emphasize that even milder stages of AKI also are associated with a worse overall survival.

This study has some limitations. First, no specific sample size calculation was performed for this sub-study, however, due to the high event rate of AKI and death in this study, we were able to perform sufficient hypothesis testing on group level. Second, we used the plasma creatinine measured upon hospital admission to establish a baseline eGFR. Even though median transport to the hospital only was 22 (10–32) min, cardiopulmonary resuscitation may theoretically have resulted in creatinine release from the skeletal muscle and thereby influenced the baseline value. Third, since we do not have reliable estimates of the individual patient's fluid balance, this may confound the observed differences in relation to creatinine values and urine output. For instance, the fluid balance may be aimed negative due to volume overload in CKRT patients, and therefore “true creatinine” could potentially have been even higher in this group, which complicates head-to-head comparisons. Fourth, patients developing AKI in ICU have an increased risk of developing chronic kidney disease later on [34], but due to the limited follow-up duration of only 365 days, this may not yet have been established, and thus the longer-term consequences are not revealed within the observation time in the present study. Lastly, this study was conducted on a relatively homogenous population with favorable clinical characteristics. Therefore, it is speculative whether results can be generalized to OHCA populations without shockable rhythm or bystander cardiopulmonary resuscitation.

In conclusion, this study of OHCA patients, found that AKI patients requiring CKRT had longer time-to-ROSC and worse metabolic derangement at hospital admission. AKI patients, with or without initiation of CKRT, had a worse 1-year survival compared to non-AKI patients. This risk persisted after adjusting for other peri-arrest risk factors.

### Supplementary Information

Supplementary Material 1.

## Data Availability

The datasets used and/or analyzed during the current study are available from the corresponding author on reasonable request.

## Abbreviations

AKI: Acute kidney injury
CKRT: Continuous kidney replacement therapy
CPR: Cardiopulmonary resuscitation
GFR: Glomerular filtration rate
KDIGO: Kidney disease: improving global outcomes-classification
MAP: Mean arterial pressure
OHCA: Out-of-hospital cardiac arrest
PaO_2_: Partial pressure of arterial oxygen
PCAS: Post-cardiac arrest syndrome
ROSC: Return of spontaneous circulation
TTM: Targeted temperature management
