# Supplementary material for: Acute kidney injury after out-of-hospital cardiac arrest
Source: Crit Care. 2024 May 18;28:169. doi: 10.1186/s13054-024-04936-w (PMC11102609; doi:10.1186/s13054-024-04936-w)
Supplement: Supplementary file 1 — Supplementary Material 1. [file 13054_2024_4936_MOESM1_ESM.pdf]

SUPPLEMENTAL MATERIAL

Acute Kidney Injury after Out-of-Hospital Cardiac Arrest

Contents

Steering Committee 2

Data Safety Monitoring Board 3

BOX-trial inclusion and exclusion criteria 4

Trial registration 5

Trial interventions.. 6

Supplementary Figure S1 - Consort Diagram 7

Supplementary Figure S2 – Perfusion pressure 8

Supplementary Table S1 - KDIGO AKI definition 9

Supplementary Table S2 - Perfusion pressure 10

Supplementary Table S3 – Proportion on continuous kidney replacement therapy (CKRT).................... 11

Supplementary Table S4 – Duration of continuous kidney replacement therapy (CKRT)......................... 12

Supplementary Table S5 - Vasoactive Inotropic Score (VIS) during hospitalization................................. 13

Supplementary Table S6 - Contrast media use.. 14

Supplementary Table S7 - Obstructive coronary artery disease... 15

Supplementary Table S8 – Kaplan Meier overall survival 30 days.. 16

Steering Committee

The trial was designed and overseen by the steering committee

Principial Investigator and sponsor

Associate Professor Jesper Kjaergaard MD PhD DMSc.

Heart Center, Department of Cardiology, Copenhagen University Hospital Rigshospitalet, Copenhagen, and Department of Clinical Medicine, University of Copenhagen, Copenhagen, Denmark

Associate Professor Henrik Schmidt MD DMSc

Department of Anesthesiology Odense University Hospital, Odense, and Department of Clinical Research, University of Southern Denmark.

Professor Jacob Eifer Møller MD PhD DMSc

Department of Cardiology, Odense University Hospital, Odense, Heart Center, Department of Cardiology, Copenhagen University Hospital Rigshospitalet, Copenhagen, and Department of Clinical Research,University of Southern Denmark.

Professor Christian Hassager MD DMSc

Heart Center, Department of Cardiology, Copenhagen University Hospital Rigshospitalet, Copenhagen, and Department of Clinical Medicine, University of Copenhagen, Copenhagen, Denmark.

Data Safety Monitoring Board

An independent data and safety monitoring committee oversaw the trial and reviewed the planned interim analysis after 400 patients had completed the 90-day follow-up. The Data Safety Monitoring Board members were:

Professor Lars Køber (Chair),

Heart Center, Department of Cardiology, Copenhagen University Hospital Rigshospitalet, Copenhagen, and Department of Clinical Medicine, University of Copenhagen, Copenhagen, Denmark

Professor Kirsten Møller,

Department of Neuro-anesthesiology, The Neuroscience Centre and Institute for Clinical Medicine, Faculty of Health Sciences, University of Copenhagen; Denmark

Clinical associate research professor Jens Jakob Thune (performing the statistical analyses for the Data Safety Monitoring Board),

Department of Cardiology, Copenhagen University Hospital Bispebjerg Hospital, and Department of Clinical Medicine, University of Copenhagen, Copenhagen, Denmark

BOX-trial inclusion and exclusion criteria

Inclusion criteria

1. Age ≥18 years
2. OHCA of presumed cardiac cause
3. Sustained ROSC#
4. Unconsciousness (GCS < 9) (patients not able to obey verbal commands) after sustained ROSC

#Sustained ROSC: Sustained ROSC is when chest compressions have been not required for 20 consecutive minutes and signs of circulation persist.

Exclusion criteria

1. Conscious patients (obeying verbal commands)
2. Females of childbearing potential (unless a negative HCG test can rule out pregnancy within the inclusion window)
3. In-hospital cardiac arrest (IHCA)
4. OHCA of presumed non-cardiac cause, e.g., after trauma or dissection/rupture of major artery OR cardiac arrest caused by initial hypoxia (i.e., drowning, suffocation, hanging)
5. Known bleeding diathesis (medically induced coagulopathy (e.g., warfarin, NOAC, clopidogrel) does not exclude the patient
6. Suspected or confirmed acute intracranial bleeding
7. Suspected or confirmed acute stroke
8. Unwitnessed asystole
9. Known limitations in therapy and Do Not Resuscitate-order
10. Known disease making 180 days survival unlikely
11. Known pre-arrest CPC 3 or 4
12. > 4 h (240 min) from ROSC to screening
13. Systolic blood pressure < 80 mmHg despite fluid loading/vasopressor and/or inotropic medication/intra-aortic balloon pump/axial flow device#
14. Temperature on admission < 30 °C

If the systolic blood pressure (SBP) is recovering during the inclusion window (220 min), the patient can be included.

Trial registration

The BOX trial has been registered at Clinicaltrials.gov. These are the dates of registration and start and conclusion of participant in the trial:

10th of March 2017: first patient was included in the trial after all approvals from Danish Authorities had been obtained.

30th of April 2017: Trial registration at clinicaltrial.gov was started. The registration was 1 month, and 20 days delayed compared to the trial initiation because of delays with the trial primary investigator.

2nd of May 2017: Trial registration published (NCT03141099). At that point in time 11 patients had been included in the trial

June 30, 2022 Completion of trial posted

Trial interventions

Blinded blood-pressure intervention: Invasive patient-specific blood-pressure modules were adapted to the trial with modification of the internal calibration to report a blood-pressure either 10% higher or 10% lower than the actual blood-pressure according to allocation of randomization. Hence, when targeting a MAP of 70 mmHg in all trial patients, half of the patients had an actual MAP treatment target of 63 mmHg, whereas the other half of the patients had an actual MAP treatment target of 77 mmHg.

Open-label oxygen intervention: Patients were randomized to a restrictive oxygen treatment with a target partial pressure of arterial oxygen (PaO_2) of 9-10 kPa or a liberal oxygen treatment with a target PaO_2 of 13-14 kPa. The allocated oxygen intervention was initiated instantly upon randomization, and maintained during mechanical respiratory support.

Targeted temperature management intervention: Patients underwent targeted temperature management at 36°C for a minimum of 24 hours followed by either a target of 37°C for 12 or 48 hours. Temperature was raised by < 0.5^{o}C/hour until normothermia.

Supplementary Figure S1 - Consort Diagram

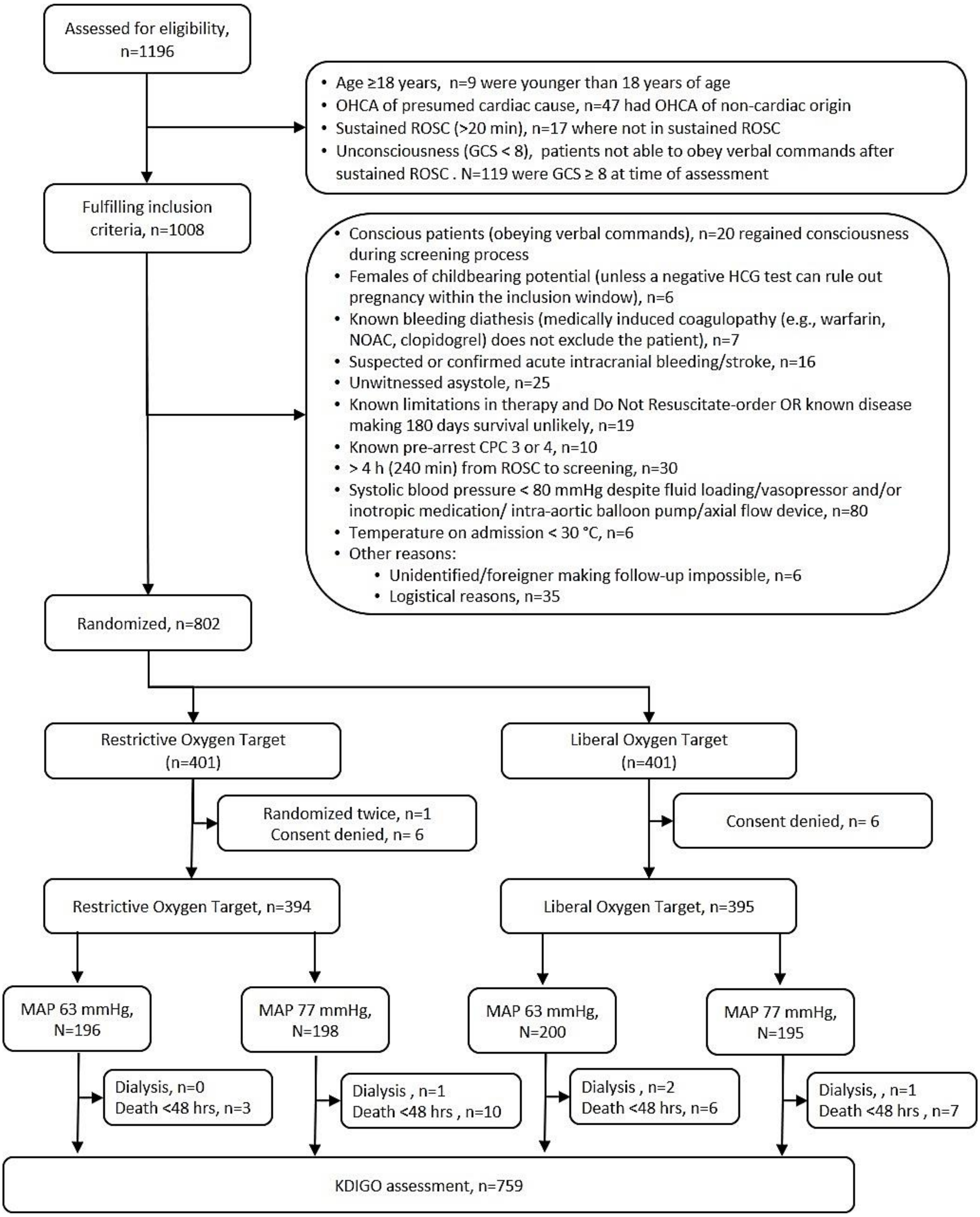

Supplementary Figure S2 – Perfusion pressure (mean arterial pressure – central venous pressure)

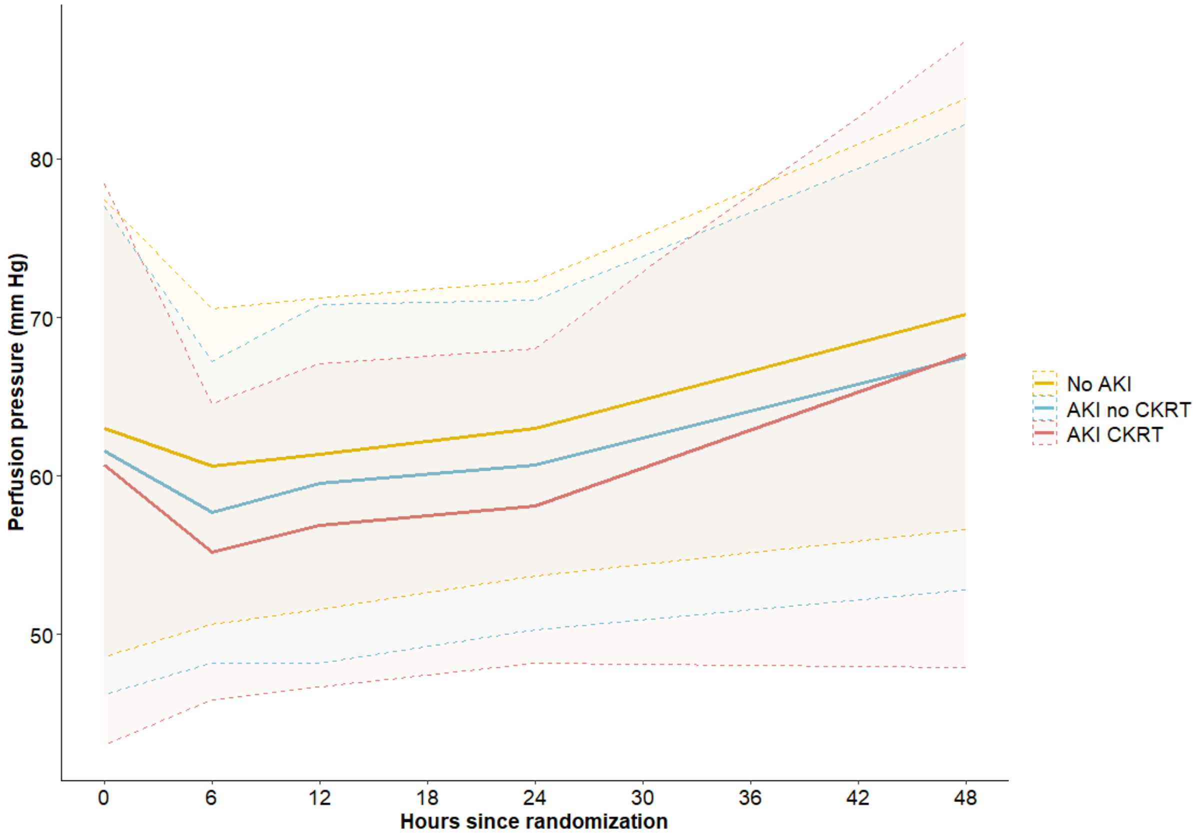

Figure S2. Mean perfusion pressure (± standard deviation) over time according to group – No AKI, AKI no CKRT, AKI CKRT. Abbreviations: *AKI;* acute kidney injury, *CKRT;* continuous kidney replacement therapy.

Supplementary Table S1 – KDIGO AKI definition

Acute kidney injury (not graded) was defined according to Kidney Disease: Improving Global Outcomes (KDIGO)[9] as any of the following:

- Increase in SCr by ≥ 0.3 mg/dl (≥ 26.5 μmol/l) within 48 hours; or
- Increase in SCr to ≥ 1.5 times baseline, which is known or presumed to have occurred within the prior 7 days; or
- Urine volume < 0.5 ml/kg/h for 6 hours.

Acute kidney injury was further staged according to the following criteria

Stage	Serum creatinine	Urine output
1	1.5–1.9 times baseline<br>OR<br>≥ 0.3 mg/dl (≥ 26.5 μmol/l) increase	< 0.5 ml/kg/h for 6–12 hours
2	2.0–2.9 times baseline	< 0.5 ml/kg/h for ≥ 12 hours
3	3.0 times baseline<br>OR<br>Increase in serum creatinine to ≥ 4.0 mg/dl (≥ 353.6 μmol/l)<br>OR<br>Initiation of renal replacement therapy	< 0.3 ml/kg/h for ≥ 24 hours<br>OR<br>Anuria for ≥ 12 hours

Supplementary Table S2– Perfusion pressure (mean arterial pressure – central venous pressure)

	**No AKI**	**AKI no CKRT**	**AKI CKRT**	*p*-value
Perfusion pressure time 0, mmHg	63 ± 14	62 ± 15	61 ± 18	0.46
Perfusion pressure 6 hours, mmHg	61 ± 10	58 ± 10	55 ± 9	**<0.001**
Perfusion pressure 12 hours, mmHg	61 ± 10	60 ± 11	57 ± 10	**0.004**
Perfusion pressure 24 hours, mmHg	63 ± 9	61 ± 10	58 ± 10	**<0.001**
Perfusion pressure 48 hours, mmHg	70 ± 15	68 ± 17	68 ± 18	0.31

Data listed as mean (± standard deviation).

Supplementary Table S3– Proportion on continuous kidney replacement therapy (CKRT)

	No CKRT	CKRT
Day 0	42 (54.5%)	35 (45.5%)
Day 1	23 (29.9%)	54 (70.1%)

Data listed as count (percentage) of the total number of patients being on CKRT during ICU. CKRT; continuous kidney replacement therapy

Supplementary Table S4– Duration of continuous kidney replacement therapy (CKRT)

	**CKRT**
One day	12 (15.6%)
Two days	14 (18.2%)
Three days	16 (20.8%)
> three days	35 (45.5%)

Data listed as count (percentage) of the total number of patients being on CKRT during ICU. CKRT; continuous kidney replacement therapy

Supplementary Table S5– Vasoactive Inotropic Score (VIS)

	**No AKI**	**AKI no CKRT**	**AKI CKRT**	*p*-value
VIS sum admission to ICU	0 (0-9)	2 (0-10)	15 (0-20)	**0.003**
VIS sum at 6 hours	10 (4-21)	10 (4-23)	26 (9-56)	**<0.001**
VIS sum at 12 hours	14 (7-25)	14 (7-27)	30 (13-63)	**<0.001**
VIS sum at 24 hours	14 (6-28)	13 (6-30)	39 (16-64)	**<0.001**
VIS sum at 36 hours	10 (2-25)	8 (1-23)	28 (10-50)	**<0.001**
VIS sum at 48 hours	7 (0-19)	9 (0-25)	20 (8-50)	**<0.001**
VIS sum at 72 hours	6 (0-16)	4 (0-14)	10 (4-28)	**0.003**
VIS sum at 96 hours	6 (0-16)	4 (0-12)	8 (1-18)	0.16

Data listed as median (25th – 75th percentile). ICU; intensive care unit, Sum; summed up, VIS; vasoactive inotropic score

Supplementary Table S6 – contrast media use

	**No AKI**	**AKI no CKRT**	**AKI CKRT**	*p*-value
CAG contrast use (ml)	40 (30-50)	40 (30-50)	35 (30-50)	0.45
PCI contrast use (ml)	60 (40-100)	50 (40-90)	40 (30-50)	0.18
Total contrast use (ml)	63 (40-100)	70 (50-100)	50 (40-62)	0.41

Data are listed as median (25th-75th percentile). Data could only be obtained for patients included at Odense University Hospital (n=273). CAG; coronary angiography, PCI; percutaneous coronary intervention.

Supplementary Table S7 – obstructive coronary artery disease

	No AKI	AKI no CKRT	AKI CKRT	*p*-value
				0.42
One vessel	61 (36.1 %)	38 (46.3 %)	7 (58.3%)	
Two vessels	31 (18.3 %)	14 (17.1 %)	2 (16.7 %)	
Three vessels	22 (13.0 %)	6 (7.3 %)	0 (0 %)	
Atheromatosis	14 (8.3 %)	10 (12.2 %)	0 (0 %)	
Normal	41 (24.3 %)	14 (17.1 %)	12 (25.0 %)	

Data listed as n/n (%). Data could only be obtained for patients included at Odense University Hospital (n=273).

Supplementary Table S8 – Kaplan Meier overall survival 30 days

Survival probability according to group			
	No AKI	*AKI no CKRT*	*AKI CKRT*
10 days	85.2 %	67.8 %	53.3 %
20 days	82.2 %	67.8 %	48.1 %
30 days	81.4 %	66.7 %	48.1 %
